# Low-density nanoporous iron foams synthesized by sol-gel autocombustion

**DOI:** 10.1186/1556-276X-7-129

**Published:** 2012-02-14

**Authors:** Zhenghe Hua, Yu Deng, Kenan Li, Shaoguang Yang

**Affiliations:** 1 National Laboratory of Microstructures, Nanjing University, Nanjing, 210093, China; 2 School of Physics and Electronic Electrical Engineering, Huaiyin Normal University, Huaian, 223300, China

**Keywords:** sol-gel autocombustion, porous, iron foam, saturation magnetization

## Abstract

Nanoporous iron metal foams were synthesized by an improved sol-gel autocombustion method in this report. It has been confirmed to be pure phase iron by X-ray diffraction measurements. The nanoporous characteristics were illustrated through scanning electron microscope and transmission electron microscope images. Very low density and quite large saturation magnetization has been performed in the synthesized samples.

## Introduction

Porous nanostructured materials possess many good properties including high surface area, ultralow density, and high strength-to-weight ratio. So they are attractive materials for use in a number of catalytic [1-3], gas-sensing [4], optical [5,6], and mechanical applications [7,8]. Many kinds of porous nanostructured metal oxide foams have been synthesized and adopted in a wide range of applications, such as V_2_O_5_, TiO_2_, SiO_2_, iron oxides, and other oxides [9-14]. Additionally, nanoporous metal foams combine with properties characteristic of metals, such as good electrical and thermal conductivity, selected catalytic activity, and malleability, resulting in its desirability for acoustical insulation, electromagnetic shielding, fuel cell, catalytic applications, and plasmatic resonance [15,16], which further distinguish the potential of bulk forms of metals. This makes the synthesis of metal foams at the forefront of materials science. Approaches used to synthesize such porous metal foam nanostructures include selective etching ('dealloying') of metal alloys [1,17], self-organization of ultrathin nanowires [18], and deposition onto porous templates via physical vapor, chemical vapor, or wet chemical routes [16,19-21]. Transition metal foams of nickel, copper, cobalt, and Ni-Cu and Ni-Co alloys have been synthesized with the controlled combustion method by Peter et al. [22]. Otherwise, the synthesis of porous iron, the most important magnetic transition metal, remains a very difficult work [12]. Tappan et al. have recently reported a cyanogel-based synthesis of macroporous refractory metals well below their melting point [23,24]. Several transition metal porous foams have been obtained by heating the cyanogel under an inert atmosphere. However, as mentioned in the references, there are several disadvantages in their synthesis metrology: firstly, the by-products in their thermal processing contain very toxic hydrogen cyanide and cyanogen; secondly, the metal complexes with energetic ligand bistetrazolamine used in the process are expensive and complex to be synthesized; and thirdly, elemental analysis confirmed that the Fe foams contain only approximately 50% Fe, and the iron foams are not perceptibly magnetic prior to heat treatment under a flow of Ar or H_2 _gas [23,25]. In this communication, we demonstrate a method, which is extended from the sol-gel autocombustion route [26], for the synthesis of nanoporous iron foams. The synthesis method is inexpensive and very convenient, while the obtained iron foams present quite large saturation magnetization at room temperature.

## Experiment

The iron foams were synthesized by an improved sol-gel autocombustion method. We have just introduced a sol-gel autocombustion method in the preparation of several metals and alloys recently [26]. However, this method met some difficulties in the synthesis of metal iron. The metal iron is more active than other metals such as Co and Ni; thus, it is more difficult to reduce the metal from the iron oxide(s) than the cobalt oxide(s) and nickel oxide(s). In this study, we found that the reduction ability of the sol-gel combustion process can be improved by the addition of a suitable amount of ethanol in the preparation of the sol; thus, the metal iron can be reduced from its dried gel by the improved sol-gel autocombustion process.

In short, a sol-gel approach was applied in the preparation by using iron nitrate (Fe(NO_3_)_3_·9H_2_O) and citric acid (C_6_H_8_O_7_·H_2_O) as the starting materials, and ethanol and distilled water (rather than only distilled water in our previous report [26]) as the dissolvent. In a typical experiment, 12.5 mmol citric acid and 10 mmol iron nitrate were dissolved in 50 ml distilled water. The solution was ultrasonic agitated for about 10 min after adding 8 ml ethanol. Then the pH value of the solution was adjusted to 5 to approximately 6 by ammonia. The resultant solution was poured into a beaker and then boiled for about 2 min by an electrical furnace to drive off the air in the solution before transferring into a baking box heated at 95°C to develop a dried gel. Then the dried gel was put into a quartz tube and washed by pure nitrogen gas for about 30 min. After that, the nitrogen gas was cut down, and then the tube with the dried gel was transferred into a tube furnace heated to the preset temperature to activate the combustion. The gel burned violently, and a large amount of gas was released. After the reaction, the product, loose iron foam, was cooled down to room temperature under the protection of nitrogen. The dried gel can be ignited at different temperatures above its ignition point (little higher than 200°C as measured below). We have synthesized several samples with different ignition temperatures of 300°C, 400°C, 500°C, 600°C, and 700°C, respectively. For comparison of magnetic properties, we further prepared a sample by annealing the 600°C-ignited product for 3 h with the protection of the hydrogen gas just after the combustion.

The resulting materials were characterized by X-ray diffraction [XRD] with CuKα radiation, scanning electron microscope [SEM], transmission electron microscope [TEM], and vibration sample magnetometer [VSM]. Thermogravimetry [TG] and mass spectrometry were applied for the analysis of the combustion of the dried gel under the protection of the argon gas.

## Results and discussion

**Figure 1:**
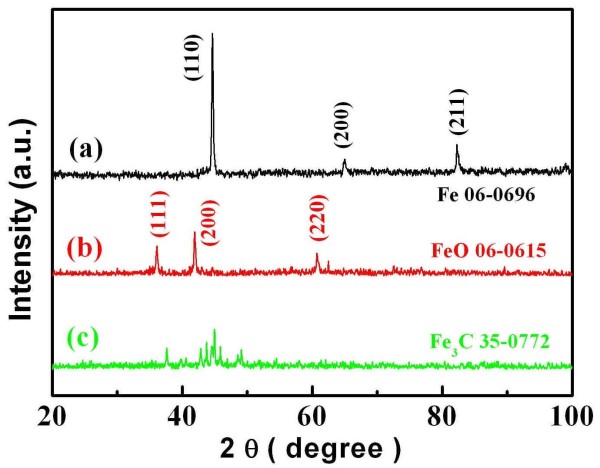
**XRD patterns of samples ignited at 600°C**. The ratios of citric acid and iron nitrate are (a) 1.25:1, (b) 1:1, and (c) 1.5:1, respectively.

The crystalline phase of the samples was determined by XRD measurements. Figure 1 shows the XRD patterns of the samples prepared with the ignition temperature of 600°C. From this pattern, it can be found that the obtained iron sample is very pure. In order to find out the suitable experiment condition, we have tried to adjust the ratio of citric acid and iron nitrate. The result showed that the ratio is of paramount importance. The excessiveness of the citric acid is just as bad as its deficiency. When the citric acid is excessive, the combustion is deficient, and the remaining carbon was left in the obtained samples. When the citric acid is deficient, the iron cannot be reduced thoroughly, and ferric oxide was obtained in the samples. As shown in Figure 1, when the molar ratios of the citric acid and iron nitrate are 1:1, 1.25:1, and 1.5:1, the obtained samples are FeO (PDF no. 06-0615), Fe (PDF no. 06-0696), and Fe_3_C (PDF no. 35-0772), respectively. Since peaks in the XRD pattern of the Orthorhombic phase were so dense, the indices of the crystallographic plane are not shown in Figure 1, pattern (c), though all the peaks can be indexed as Fe_3_C.

The moderate addition of ethanol is as important as the ratio of citric acid and iron nitrate. We have tried many synthesis parameters and found that, without the ethanol in the preparation of the sol, no iron peaks can be observed in the XRD patterns of the synthesized samples. Although the detailed reason is not clear, the ethanol plays a very important role in the synthesis of iron foams. All the samples mentioned below are obtained with the ratio (citric acid, iron nitrate, and ethanol) of 12.5 mmol:10 mmol:8 ml.

The obtained silvery gray Fe foams are very active and can even burn violently in air and form brownish red powder because of the dumping friction during collection from the quartz tube. The samples can be easily attracted by a magnet, while the burned brownish red powder cannot. This may reveal the formation of the iron with very fine particles in the combustion synthesis of gels, while the brownish red antiferromagnetic α-Fe_2_O_3 _formed in the burning of the produced iron.

**Figure 2:**
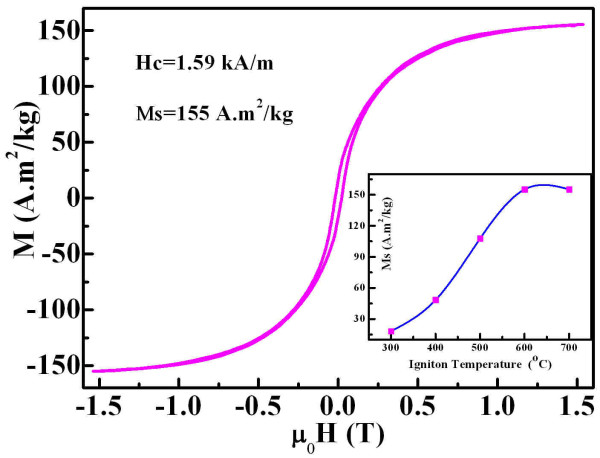
**Room temperature magnetic hysterisis loop of the 600°C-ignited sample**. A clear loop can be observed in the VSM result which reveals ferromagnetism of the sample. The saturation magnetization is about 155 A·m^2^/kg, and the coercivity is about 1.59 kA/m. Inset shows the relationship between saturation magnetization of the prepared sample and the ignition temperature.

The magnetic properties of the synthesized samples have been studied by VSM at room temperature. Hysterisis loops [M-H] can be obtained for all samples ignited at different temperatures. It is found that the saturation magnetization reaches the highest value at the ignition temperature of 600°C. Figure 2 shows the M-H loop of the 600°C-ignited sample with the inset of the relationship between the saturation magnetization and the ignition temperature. The sample is almost saturated at 1.5 T. The saturation magnetization is about 155 A·m^2^/kg, and the coercivity is about 1.59 kA/m. Two main possible impurities, α-Fe_2_O_3 _and Fe_3_O_4_, are expected in the obtained samples. To our knowledge, α-Fe_2_O_3 _is an antiferromagnetic material, and Fe_3_O_4 _is a ferrimagnetic material with the saturation magnetization of about 92 A·m^2^/kg, while the saturation magnetization of the bulk iron is about 217 A·m^2^/kg, which is quite larger than that of the Fe_3_O_4_. For nanostructured magnetic particles, the saturation magnetization is smaller than that in their bulk state because of the oxidation of metals at very large surface and superparamagnetism in very small particles. The measured saturation magnetization of high-temperature-ignited samples is very large, which reveals that the purity of the synthesized iron is quite good. The experiment shows that the saturation magnetization of the samples can be improved a little and reach about 165 A·m^2^/kg by annealing in hydrogen. It is conjectured that two processes may occur in the hydrogen annealing: one is the reduction of the remaining iron oxide(s) in the sample, and another is the growth of the iron nanoparticles produced in the combustion. By comparing the VSM results, it can be concluded that the purity of the high-temperature-ignited samples is quite good although they are synthesized by only simple combustion.

**Figure 3:**
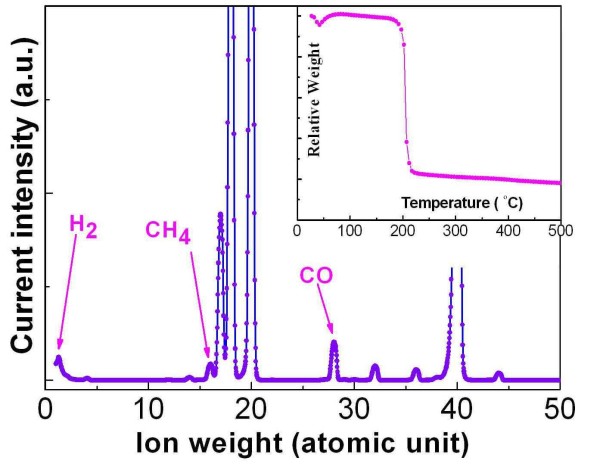
**Mass spectrum of the released gas near the combustion temperature of the dried gel**. From this result, the reducing gases H_2_, CH_4_, and CO can be observed clearly. Inset shows the TG measurement result of the dried gel. The rapid decrease of the weight of the dried gel reveals the combustion temperature near 200°C.

In order to understand the synthesis mechanism in the combustion, thermogravimetry and mass spectrometry were applied for the analysis of the combustion of the dried gel under the protection of the argon gas. Figure 3 shows the mass spectrum recorded near the ignition temperature with the inset of the TG measurement result. It can be found that, except for the H_2_O, NH_3_, CO_2_, and the protection gas Ar, three kinds of reducing gases (as marked by arrows in Figure 3), H_2_, CH_4_, and CO, have been detected in the released gas of the combustion. The reduction ability of CO, CH_4_, and H_2 _is very strong in their nascent state. Although the detailed process is not very clear, the metal iron should be reduced from the iron oxide(s) decomposed from the dried gel by CO, CH_4_, and H_2 _in the combustion. As there is a large amount of gas released in the combustion, the synthesized samples should posses a porous structure in the final form. From the TG measurement result, it can be found that the weight of the gel decreases very rapidly near 200°C, which means that the dried gel can be ignited a little higher than 200°C. In fact, we have noticed that the dried gel can even be ignited by a cigarette lighter. To control the ignition temperature easily, we use a tube furnace as the activation source, which not only supplies the ignition but also controls the circumstance temperature of the resulting samples. Known to us all, the temperature can rise to higher than 1,000°C in the combustion and then decrease very rapidly to the temperature of its surroundings. Similar to the previous reports, some impurities will form in the production of iron foams [23]. When the surrounding temperature is too low, the scale of the Fe particles will be very small, and superparamagnetism will exist. Thus, the saturation magnetization of the samples prepared at low temperature is very small, while the higher surrounding temperature will make the produced metal iron particles grow and help improve the purity. Thus, the saturation magnetization of the corresponding samples is larger. This is consistent with our magnetic studies quite well.

**Figure 4:**
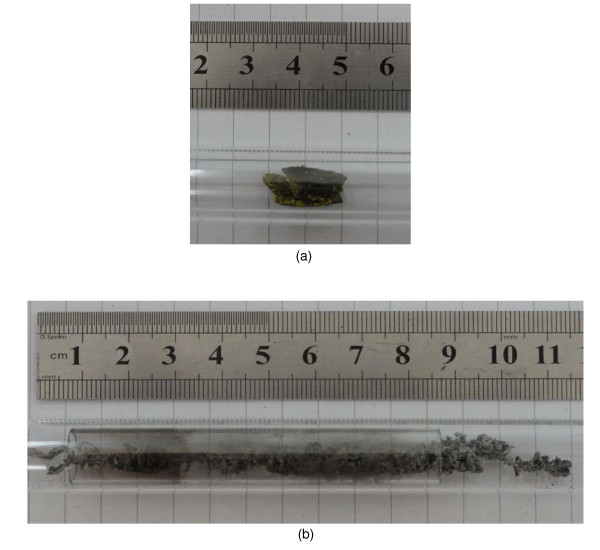
**Photographs before and after combustion**. (**a**) Photograph of the gel in a quartz tube before combustion. (**b**) Photograph of the porous iron foam after combustion. The volume expanded greatly compared with the corresponding gel before combustion. The color is silver gray, and the foam can be attracted by a magnet.

**Figure 5:**
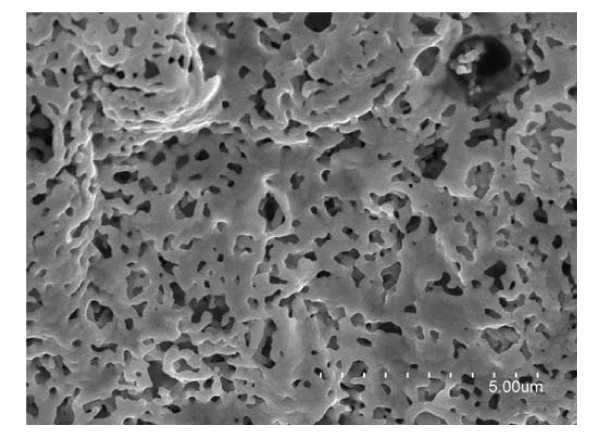
**SEM image of the 600°C-ignited sample**. Nano-porous structure can be observed.

The volume increased greatly in the combustion. From the photographs shown in Figure 4a,b, it can be seen clearly that the volume of the synthesized sample expanded about ten times larger than that of the corresponding gel. This volume expansion reveals the very low density and porous structure of the synthesized samples. Large amount of gas released in the combustion should be the reason of the formation of the porous structures. The morphology of the sample was characterized by SEM. Figure 5 shows a typical SEM image of the sample ignited at 600°C. From this image, many pores can be observed with the diameter of 50 to approximately 500 nm. This reveals that the produced sample is porous, and thus, the measured density of the sample is as low as 0.002 g/cm^3^. This nanoporous structured metal has the advantage of the penetration of gases, high thermal conductance, and high electric conductance.

**Figure 6:**
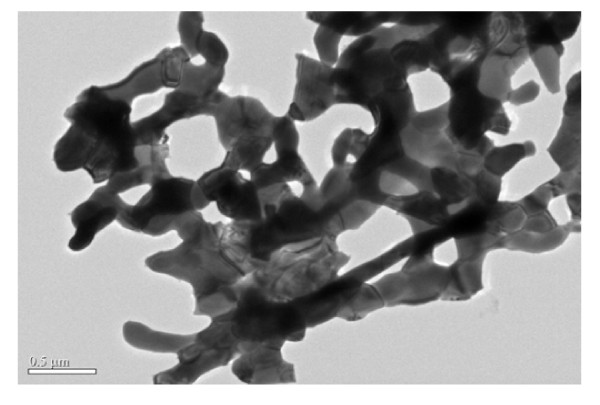
**Typical TEM image of the sample ignited at 600°C**. Nanoporous structure is formed through the connection of nanoparticles with the dimension of about 200 nm in width and 600 nm in length.

**Figure 7:**
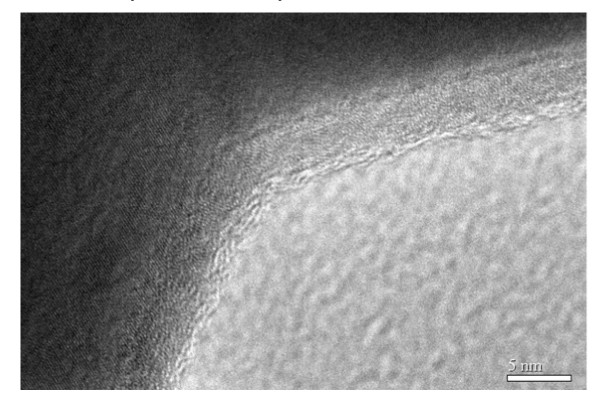
**High-resolution TEM image of a particle**. The distance between the fringes was measured to be about 0.202 nm, which is in accordance with that of the (110) planes of the iron.

TEM was applied in the further study of the structures of the samples. Figure 6 shows a typical TEM image of the 600°C-ignited sample. From this image, porous structure is seen to be formed by the connection of nanoparticles with the dimension of about 200 nm in width and 600 nm in length. High-resolution TEM study was also performed for the sample. As shown in Figure 7, the distance between two neighboring fringes in the HRTEM image is about 0.202 nm, which is in accordance with that of the (110) planes of the iron. This further confirms the formation of the metal iron in the combustion. There are many pores among the iron particles, which make the sample loose and very low in density.

## Conclusion

A convenient sol-gel autocombustion method was employed in the synthesis of iron foams. Moreover, the iron foams are characterized by very low density and quite large saturation magnetization at room temperature. The XRD and VSM measurements illustrated the formation of the metal iron in the combustion. SEM and TEM studies confirmed the nanoporous structure of the samples. This kind of ferromagnetic porous iron foams may find wide applications in the fields of catalysis, fuel cells, hydrogen storage, unique insulation, and electromagnetic absorption.

## Competing interests

The authors declare that they have no competing interests.

## Authors' contributions

ZH fabricated the samples, carried out the magnetic property, XRD, and density measurements. YD carried out the SEM and TEM studies. KL participated in the fabrication of the samples. SY designed the whole study, carried out the TG-Mass measurements, participated in the SEM and TEM studies, analyzed the data, and prepared the manuscript. All authors read and approved the final manuscript.

## References

[B1] Zielasek V Juergens B Schulz C Biener J Biener MM Hamza AV Baumer M Gold catalysts: nanoporous gold foams Angew Chem Int Ed 2006 45 8241 8244 10.1002/anie.200602484 17099919

[B2] Patel AC Li S Wang C Zhang W Wei Y Electrospinning of porous silica nanofibers containing silver nanoparticles for catalytic applications Chem Mater 2007 19 1231 1238 10.1021/cm061331z

[B3] Xu J White T Li P He CH Han YF Hydroxyapatite foam as a catalyst for formaldehyde combustion at room temperature J Am Chem Soc 2010 132 13172 13173 10.1021/ja1058923 20815345

[B4] Liu Z Searson PC Single nanoporous gold nanowire sensors J Phys Chem B 2006 110 4318 4322 10.1021/jp056940t 16509729

[B5] Kucheyev SO Hayes JR Biener J Huser T Talley CE Hamza AV Surface-enhanced Raman scattering on nanoporous Au Appl Phys Lett 2006 89 053102 10.1063/1.2260828

[B6] Wei G Wang L Liu Z Song Y Sun L Yang T Li Z DNA-network-templated self-assembly of silver nanoparticles and their application in surface-enhanced Raman scattering J Phys Chem B 2005 109 23941 23947 10.1021/jp054752x 16375382

[B7] Weissmuller J Viswanath RN Kramer D Zimmer P Wurschum R Gleiter H Charge-induced reversible strain in a metal Science 2003 300 312 315 10.1126/science.1081024 12690195

[B8] Biener J Hodge AM Hamza AV Hsiung LM Satcher JH Nanoporous Au: a high yield strength material J Appl Phys 2005 97 024301 10.1063/1.1832742

[B9] Chandrappa GT Steunou N Livage J Macroporous crystalline vanadium oxide foam Nature 2002 416 18 702 702 11961545 10.1038/416702a

[B10] Arabatzis IM Falara P Synthesis of porous nanocrystalline TiO2 foam Nano Lett 2003 3 2 249 251 10.1021/nl0259028

[B11] Carn F Saadaoui H Masse P Ravaine S Julian-Lopez B Sanchez C Deleuze H Talham DR Backov R Three-dimensional opal-like silica foams Langmuir 2006 22 5469 5475 10.1021/la060220b 16732679

[B12] Deshpande K Mukasyan A Varma A Direct synthesis of iron oxide nanopowders by the combustion approach: reaction mechanism and properties Chem Mater 2004 16 4896 4904 10.1021/cm040061m

[B13] Venugopal BR Samuel EP Shivakumara C Rajamathi M Macroporous metal oxide foams through self-sustained combustion reactions J Porous Mater 2009 16 205 208 10.1007/s10934-008-9186-y

[B14] Drisko GL Zelcer A Luca V Caruso RA de AGJ Soler-Illia A One-pot synthesis of hierarchically structured ceramic monoliths with adjustable porosity Chem Mater 2010 22 4379 4385 10.1021/cm100764e

[B15] Dixon MC Daniel TA Hieda M Smilgies DM Chan MHW Allara DL Preparation, structure, and optical properties of nanoporous gold thin films Langmuir 2007 23 2414 2422 10.1021/la062313z 17249701

[B16] Biener J Nyce GW Hodge AM Biener MM Hamza AV Maier SA Nanoporous plasmonic metamaterials Adv Mater 2008 20 1211 1217 10.1002/adma.200701899

[B17] Erlebacher J Aziz MJ Karma A Dimitrov N Sieradzki K Evolution of nanoporosity in dealloying Nature 2001 410 450 453 10.1038/35068529 11260708

[B18] Liu R Liu JF Yu SJ Liu Q Jiang GB Capping agent replacement induced self-organization of ultrathin nanowires: a new and general approach for fabricating noble metal nanoporous films with small ligament sizes Chem Commun 2001 47 1613 1615 10.1039/c0cc04490c 21109902

[B19] Walsh D Arcelli L Ikoma T Tanaka J Mann S Dextran templating for the synthesis of metallic and metal oxide sponges Nat Mater 2003 2 386 390 10.1038/nmat903 12764358

[B20] Haupt M Miller S Glass R Arnold M Sauer R Thonke K Moller M Spatz JP Nanoporous gold films created using templates formed from self-assembled structures of inorganic-block copolymer micelles Adv Mater 2003 15 829 831 10.1002/adma.200304688

[B21] Bao ZH Ernst EM Yoo S Sandhage KH Syntheses of porous self-supporting metal-nanoparticle assemblies with 3D morphologies inherited from biosilica templates (diatom frustules) Adv Mater 2009 21 474 478 10.1002/adma.200801499

[B22] Erri P Nader J Varma A Controlling combustion wave propagation for transition metal/alloy/cermet foam synthesis Adv Mater 2008 20 1243 1245 10.1002/adma.200701365

[B23] Tappan BC Huynh MH Hiskey MA Chavez DE Luther EP Mang JT Son SF Ultralow-density nanostructured metal foams: combustion synthesis, morphology, and composition J Am Chem Soc 2006 128 6589 6594 10.1021/ja056550k 16704258

[B24] Tappan BC Steiner SA III Luther EP Nanoporous metal foams Angew Chem Int Ed 2010 49 4544 4565 10.1002/anie.200902994 20514651

[B25] Burgess CM Vondrova M Bocarsly AB A versatile chemical method for the formation of macroporous transition metal alloys from cyanometalate coordination polymers J Mater Chem 2008 18 3694 3701 10.1039/b804258f

[B26] Jiang YW Yang SG Hua ZH Huang HB Sol-gel autocombustion synthesis of metals and metal alloys Angew Chem Int Ed 2009 48 8529 8531 10.1002/anie.200903444 19798710

